# A lung cancer mouse model system based on an inbred C3H strain: Ultrasound imaging, pathological analysis, and proteomic biomarker identification

**DOI:** 10.14202/vetworld.2025.1101-1108

**Published:** 2025-05-12

**Authors:** Ulayatul Kustiati, Wahyu Tri Widayati, Dwi Liliek Kusindarta, Dwi Aris Agung Nugrahaningsih, Dinda Aliffia, Wilda Bunga Tina Sanjaya, Hevi Wihadmadyatami

**Affiliations:** 1 Department of Anatomy, Faculty of Veterinary Medicine, Universitas Gadjah Mada, Yogyakarta, Indonesia, Faculty of Veterinary Medicine, Universitas Gadjah Mada, Yogyakarta, Indonesia; 2 Laboratory of Pharmacology, Faculty of Veterinary Medicine, Universitas Brawijaya, Malang, East Java, Indonesia; 3 Department of Pathology Anatomy, Regional General Hospital of Karanganyar Regency, Karanganyar, Central Java, Regional General Hospital of Karanganyar Regency, Karanganyar, Central Java; 4 Department of Pharmacology and Therapy, Faculty of Veterinary Medicine, Universitas Gadjah Mada, Yogyakarta, Indonesia

**Keywords:** animal model, C3H mice, lung cancer, pathology, proteomics, ultrasound imaging

## Abstract

**Background and Aim:**

Lung cancer remains a leading cause of global mortality, necessitating robust animal models for research into its mechanisms and therapeutic options. This study aimed to develop and validate a novel lung cancer mouse model using the inbred C3H strain through intraperitoneal (I.P) injection of benzo(a)pyrene, offering insights into hematology, pathology, imaging, and proteomic biomarkers.

**Materials and Methods:**

Twelve male inbred C3H mice were assigned to non-treated and treatment groups, with the latter receiving 100 mg/kg body weight of benzo(a)pyrene intraperitoneally. Tumor development was monitored for 15 days using hematological analysis, ultrasound imaging (Vevo F2), histopathological assessment, and proteomic profiling through liquid chromatography-high-resolution mass spectrometry.

**Results:**

Hematological analysis indicated a decrease in white blood cells, lymphopenia, and neutropenia, while red blood cells, hemoglobin, and platelets remained within normal ranges. Ultrasound imaging revealed tumor formation as hypoechoic areas with irregular patterns on the lung surface. The histological analysis highlighted lymphocyte infiltration, alveolar wall thickening, fibroelastosis, and dysplastic changes in the bronchial epithelium. Proteomic profiling identified specific biomarkers associated with lung cancer, including A disintegrin and metalloproteinase with thrombospondin motifs 12, abnormal spindle, adducin-3, adhesion G protein-coupled receptor, Agrin, apoptotic chromatin condensation inducer 1, rapidly accelerated fibrosarcoma isoforms B oncogene, breast cancer gene 2, hypoxia-induced gene-1, leucine-rich repeat-containing 2, leucine-rich repeat kinase 2, leucine-rich repeat-containing protein 2 isoform X2, membrane-spanning 4-domains, subfamily A, proto-oncogene tyrosine-protein kinase Src, rat sarcoma virus-related protein 14, squamous cell carcinoma antigen, and transcription intermediary factor 1-alpha.

**Conclusion:**

The I.P administration of benzo(a)pyrene in C3H mice effectively induced lung cancer, demonstrating significant pathological and biomarker changes. This model provides a valuable platform for investigating lung cancer mechanisms, evaluating new therapeutic approaches, and potentially shortening the timeframe required to establish reliable animal models for preclinical studies.

## INTRODUCTION

Recently, lung cancer has been declared the primary cause of global health challenges and is currently the primary cause of mortality in men. A previous study by Budnik *et al*. [1] on lung cancer in humans has identified multiple risk factors, including smoking, environmental pollutants, and genetic predisposition, as major causative factors. Animal models, especially rodent models, such as rats and mice, are widely employed to understand lung cancer mechanisms and pathophysiology as well as to evaluate potential therapeutic agents [2]. There are two forms of lung cancer in humans: Small-cell lung carcinoma and non-small cell lung carcinoma (NSCLC). NSCLC consists of two subtypes: Adenocarcinoma, large cell carcinoma, and squamous cell carcinoma (SCC). NSCLC accounts for approximately 85% of lung cancer cases [3]. Only 20% of lung cancer patients survive the disease for ≥5 years. This condition arises from the common discovery of advanced lung cancer and its aggressive nature of the disease [4]. The molecular mechanisms of lung cancer involve complex interactions among genetic mutations, epigenetic changes, and changes in signaling pathways, which contribute to tumor initiation, progression, and metastasis.

Rodent animal models, including mice and rats, are crucial for lung cancer research. These models allow for investigating tumorigenesis and the evaluation of new therapeutic agents. For example, previous studies by Tanaka *et al*. [5] and Tan *et al*. [6] have demonstrated that administering carcinogens, such as benzopyrene, to rodents can induce lung tumors that are extremely similar to human lung cancer. One of the strains of inbred mice that are often used to create cancer models is C3H. C3H mice, especially male mice, tend to develop liver tumors spontaneously in adulthood [7]. C3H mice are well known and have been used as a model of spontaneous hepatocellular carcinoma (HCC) [8, 9]. C3H mice are also often employed as models of chemically induced HCC, such as the HCC model induced by diethyl nitrosamine, phenobarbital, and carbon tetra-chloride [10]. However, to date, the use of C3H mice as an animal model of lung cancer remains limited relative to that of other inbred strains, namely C57BL6.

Furthermore, related to the carcinogenesis process, to investigate the carcinogenesis process and therapeutic methods in the right animal model, information related to pathological changes and the creation of biomarker profiles, including tumor proto-oncogenes, will be extremely helpful in understanding the mechanisms involved. However, to date, no study has specifically provided information on pathology, biomarker profiles, and the identification of lung cancer growth using ultrasound imaging in lung cancer animal models, especially C3H-strain mice. This study aimed to analyze the changes in lung pathology in lung cancer animal models to identify biomarkers through protein profiling and to provide supporting data for confirming lung cancer animal models through ultrasound imaging.

## MATERIALS AND METHODS

### Ethical approval

This study received ethical clearance from the Ethics Committee of the Faculty of Veterinary Medicine, Universitas Gadjah Mada (Ethics Approval Number: 00053/EC-FKH/Int/2021).

### Study period and location

This study was conducted during June and July 2024. Experimental animal maintenance and analysis were facilitated by the Laboratory of Research and Testing of Universitas Gadjah Mada.

### Animal model experiments

Twelve male C3H mice (8 weeks old, approximately 24 g) obtained from BioLASCO (Lasco Biotech, Kangning, Taiwan) were used in this study. The mice were housed in polycarbonate cages (Techniplast, Buguggiate, Italy) under controlled conditions, including a 12-h light-dark cycle, a temperature of ±24°C, and humidity of ±70%. They had free access to food (Rat Bio, Northern Jakarta, Indonesia) and water. The mice were randomly divided into two groups: A non-treated control group and a treatment group, which received an intraperitoneal (I.P) injection of 100 mg/kg body weight (BW) of benzo(a)pyrene.

### Hematological analysis

Blood samples were collected on days 0 and 9. The ethylenediaminetetraacetic acid (Sigma-Aldrich, Massachusetts, USA)-treated blood samples underwent hematological analysis, including assessments of white blood cells (WBC), red blood cells (RBC), mean corpuscular hemoglobin, mean corpuscular volume, mean corpuscular hemoglobin concentration (MCHC), platelets (PLT), hemoglobin (HGB), lymphocytes (LYMs), and neutrophils using a Sysmex KX-21 Hematology Analyzer (Sysmex, Tampines, Singapore).

### Sample preparation for pathological analysis

On day 9, mice were euthanized using a high-dose I.P combination of 10% ketamine (100 mg/kg BW) and 2% xylazine (10 mg/kg BW) (Kepro, Maagdenberg street, Holland). Lung tissue was collected by incising the abdomen to the sternum, opening the thorax, and removing the lungs at the distal bifurcation of the trachea. The lung samples were fixed in 4% paraform-aldehyde (Nacalai Tesque, Kyoto, Japan) and processed for paraffin embedding. Tissue sections (5 μm) were stained with hematoxylin and eosin (H&E).

### Histological analysis

Deparaffinized lung sections were rehydrated with absolute ethanol (KgaA, Darmstadt, Germany), stained with hematoxylin (Bio-Optica, Milan, Italy) for 2 min, and eosin for 10 min. The sections were then dehydrated, cleared with xylol, and mounted using Canadian balsam (KgaA). The slides were examined under a light microscope (Olympus-BX51, Tokyo, Japan), and images were captured with Optilab (Yogyakarta, Indonesia).

### Ultrasound imaging

Lung cancer progression was monitored using the Vevo F2 Ultrasound System (Fujifilm, Tokyo, Japan) with a 71–30 MHz transducer. Mice were anesthetized and positioned ventrodorsally on the Vevo^®^ Imaging Station (Fuji Film, Kanagawa, Japan). After preparing the thorax and applying ultrasound gel, imaging was conducted. Changes in lung tissue were identified as hypoechoic areas with irregular patterns, and measurements were performed using the system’s features.

### Proteomics sample preparation

Blood plasma (250 μL) was mixed with 8M Urea (6 μL) and 10 mM dithiothreitol (250 μL) in 50 mM ammonium bicarbonate (Merck, Darmstadt, Germany) for 1 h at 30°C. The protein pellet was resuspended, digested with Pierce Trypsin Protease MS-grade (Thermo Fisher, Massachusetts, USA), and prepared for injection by adding 1% formic acid and filtering through 0.2 μL polyvinylidene fluoride.

### Untargeted proteomic screening (liquid chromatography-high resolution mass spectrometry [LC-HRMS])

Proteomic analysis was conducted using an Orbitrap High-Resolution Mass Spectrometer (Thermo Scientific Q Exactive, Bremen, Germany) coupled with liquid chromatography (Vanquish UHPLC Binary Pump, Thermo Scientific, Massachusetts, USA). The column used was Thermo Scientific Acclaim PepMap 100 C18 (Thermo Fisher, Vilnius, Lithuania). Untargeted protein profiling was performed using SequestHT software in Proteome Discoverer^®^ (Thermo Scientific, California, USA), with protein mapping through UniProt (https://www.uniprot.org/).

## RESULTS

### Hematological analysis

The response to I.P. administration of benzo(a)pyrene was determined using routine blood tests. The blood hematology profile of the treatment group displayed normal levels of RBC, HGB, PLT, and MCHC, indicating no anemia. However, there were lower-than-normal WBC counts with neutropenia and lymphopenia (Table 1).

**Table 1:** Hematological analysis in lung cancer animal model (inbred mice strain C3H).

Parameter	Unit	Non-treated	Treatment model	Conclusion
WBC	10^3^/µL	7.20 ± 0.30	4.65 ± 0.07	Decreases
RBC	10^6^/µL	6.44 ± 0.95	6.75 ± 0.09	Normal
HGB	g/dL	10.97 ± 1.46	11.05 ± 0.35	Normal
PLT	10^3^/µL	1045.67 ± 62.32	933.50 ± 89.80	Normal
MCV	fL	57.00 ± 2.59	54.35 ± 0.64	Decrease
MCH	Pg	17.03 ± 0.38	19.40 ± 0.28	Increase
MCHC	g/dL	29.93 ± 0.81	30.15 ± 0.92	Normal
LYM	10^3^/µL	4.60 ± 0.26	3.35 ± 0.07	Decrease
NEUT	10^3^/µL	2.60 ± 0.11	1.30 ± 0.04	Decrease

WBC=White blood cells, RBC=Red blood cells, HGB=Hemoglobin, PLT=Platelets, MCV=Mean corpuscular volume, MCH=Mean corpuscular hemoglobin, MCHC=Mean corpuscular hemoglobin concentration, LYM=Lymphocytes, NEUT=Neutrophils

### Ultrasound imaging alterations

**Figure 1:**
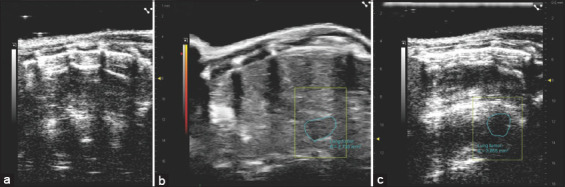
Visualization of 2D ultrasound scans of the lungs of inbred strain induced through intraperitoneal injection of benzo(a)pyrene. (a) A 2D ultrasound scan of normal mice indicated the difference between hyperechoic rib bones and anechoic lung tissues. (b) 2D ultrasound scan of the mouse chest after 7 days of benzo(a)pyrene injection displayed the presence of hypoechoic areas (highlighted in yellow) with an area measurement of 2,735 mm^2^. (c) Visualization of benzo(a)pyrene-induced lung changes after 15 days exhibited the presence of hypoechoic areas on a 2D ultrasound scan (highlighted in yellow), with the hypoechoic area growth reaching 3,855 mm^2^.

On the lung parenchyma’s brightness (B)-mode acquisition, newly developing tumors were indicated as B-lines, which indicate a tumor that formed on the mouse lung’s left surface. The developed tumors were viewed as deep clefts inside the lung parenchyma with a well-defined circumference as they grew over time. Irregular and hypoechoic area measurements suggested a significant alteration in the area (Figure 1).

### Histological alteration

**Figure 2:**
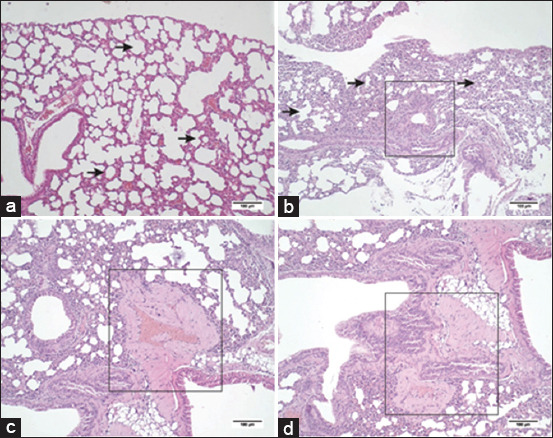
Histopathological features of the inbred mouse strain C3H induced by benzo(a)pyrene intraperitoneally. (a) Non-treated mouse lung; (b–d) treatment model mouse lung. Histopathological features show thickening of the alveoli wall (arrow), dysplastic epithelium (rectangle) (b), fibroelastosis (rectangle) between the alveoli (c), and lymphocyte infiltration (d). The examination was conducted using hematoxylin and eosin staining; Scale bar = 100 μm.

The histopathological analysis of lung tissues by H&E staining revealed several features, such as the thickening of the alveolar walls in both the non-treated and treatment model groups. In the treatment model group, LYM infiltration was observed in the tissues, with the most characteristic changes being abnormal alterations in the bronchial epithelium and increased connective tissues (fibrin and elastin) between the alveolus (Figure 2 and Table 2).

**Table 2:** Pathological changes in lung cancer animal model (inbred mice strain C3H).

Types of changes	Non-treated	Treatment model
Lymphocyte infiltration	-	+
Inflammation	-	+
Dysplastic epithelium	-	+
Adenocarcinoma	-	-
Squamous cell carcinoma	-	-
Fibroblast (Fibroelastosis)	-	+
Elastin (Fibroelastosis)	-	+
Alveoli wall thickness	-	+

- = undetected, + = detected

### Profiling of lung cancer biomarkers

Untargeted LC-HRMS using blood plasma revealed the expression of specific proteins for the formation of lung cancer that were not found in normal ani-mals, namely A disintegrin and metalloproteinase with thrombospondin motifs (ADAMTS) 12, abnormal spindle, adducin 3 (gamma), adhesion G protein-coupled receptor (aGPCRs), agrin, apoptotic chromatin condensation inducer 1 (ACIN1), B-Raf oncogene (BRAF), breast cancer gene 2 (BRCA2), hypoxia-induced gene 1, leucine-rich repeat containing 2 (LRRC2), leucine-rich repeat kinase 2 (LRRK2), leucine-rich repeat-containing protein 2 isoform X2 (LRCH2), membrane-spanning 4-domains, subfamily A (MSS4A), proto-oncogene tyrosine-protein kinase Src, rat sarcoma virus-related protein 14 (RAB14), member rat sarcoma virus (RAS) oncogene family, SCC antigen (SCCA), and transcription intermediary factor 1-alpha (TIF1α) (Table 3).

**Table 3:** Untargeted proteomic identification of blood plasma samples in lung cancer animal model (inbred mice strain C3H).

Protein	Coverage (%)	Peptides	MW (kDa)	Non-treated	Treatment model
1-acylglycerol-3-phosphate O-acyltransferase3	7	1	35	✓	✓
220-kDa myosin light chain kinase	0	1	213.7	✓	✓
A disintegrin–metallopeptidase domain 12	4	1	62.6		✓
A disintegrin and metallopeptidase domain 3 (cyritestin)	3	1	91.5	✓	✓
Abhydrolase domain containing four ABHD4	14	1	21.1	✓	✓
Abnormal spindle	1	1	363.9		✓
Adaptor-related protein	5	1	52.4	✓	✓
Adducin 3 (gamma)	4	1	76.3		✓
Adhesion G protein-coupled receptor	35	1	8.8		✓
Adhesion G protein-coupled receptor F5	3	1	126.4	✓	✓
ADP-ribosylation factor-like protein 6	11	1	22.2	✓	✓
Agrin	3	2	198.1		✓
Alpha-globin	22	3	15.1	✓	✓
Apoptotic chromatin condensation inducer 1	2	1	143.6		✓
Arylsulfatase K	53	1	5.3	✓	✓
AT-rich interactive domain-containing protein	2	1	144.3	✓	✓
ATP-binding cassette (transporter)	6	2	137.3	✓	✓
B-raf oncogene	4	1	73.5		✓
Bassoon	1	1	418.5	✓	✓
Breast cancer gene 2-interacting transcriptional	2	1	136.8		✓
Bromodomain	4	1	75.3	✓	✓
Cadherin epidermal growth factor laminin G seven-pass G-type receptor	1	1	358.2	✓	✓
Voltage-dependent calcium channel	1	1	239	✓	✓
Calmodulin-regulated spectrin-associated protein	34	1	7.8	✓	✓
Caspase recruitment domain family	2	1	114.3	✓	✓
Chloride channel protein	3	1	94.8	✓	✓
Cilia- and flagella-associated proteins	3	1	72.1	✓	✓
Collagen, type IV, alpha 5	6	1	77.9	✓	✓
Cortactin binding protein 2	2	1	123.9	✓	✓
Cyclin-dependent kinase 4	9	1	28	✓	✓
Eph receptor A1	2	1	104.8	✓	✓
Fibrillin	1	1	312.1	✓	✓
Fibronectin	1	1	253	✓	✓
Gamma-tubulin complex component 2 isoform X1	3	1	73.5	✓	✓
Hypoxia-induced gene 1 expression	26	1	10.4		✓
Inositol	1	1	195.8	✓	✓
Kallikrein-related peptidase 5	9	1	31.8	✓	✓
Kinesin-like proteins	1	1	199.8	✓	✓
L-type voltage-gated calcium channel (VGCCs/CaVs)	1	1	245	✓	✓
Latent transforming growth factor beta	1	1	186.7	✓	✓
Leucine-rich repeat containing 2	6	1	41.2		✓
Leucine-rich repeat kinase 2	1	1	284.6		✓
Leucine-rich repeat transmembrane protein coiled-coil domain containing 168	0	1	746.9	✓	✓
Leucine-rich repeat-containing protein 2 isoform X2	5	1	42.9		✓
Membrane-spanning 4-domains, subfamily A 3	13	1	17.8		✓
Microtubule-associated protein	14	1	18.2	✓	✓
Moesin	4	1	66.4	✓	✓
Nicotinamide adenine dinucleotide dehydrogenase	6	1	51.8	✓	✓
Potassium voltage-gated channel	2	1	111.4	✓	✓
Proto-oncogene tyrosine-protein kinase Src	4	1	60.6		✓
RAB14, an RAS oncogene family member	12	1	24.5		✓
Squamous cell carcinoma antigen	4	1	44.5		✓
Transcription intermediary factor 1-alpha	2	1	110.1		✓

MW=Molecular weight, ABHD4=Abhydrolase domain containing 4, ADP=Adenosine diphosphate

## DISCUSSION

The development of lung cancer animal models is essential for comprehensively understanding the mechanisms and pathophysiology of lung cancer and facilitating the discovery of new therapeutic agents. These models serve as a critical bridge between preclinical research and clinical trials, enabling the effective translation of research findings into potential treatments. This study focused on establishing a lung cancer model using I.P injection of benzo(a)pyrene in C3H-strain mice. Although the C3H strain is less commonly used than the C57BL6 strain, existing studies have demonstrated its significant role in elucidating lung cancer development mechanisms. For instance, C3H mice have been shown to develop lung tumors following specific genetic alterations, such as anaplastic lymphoma kinase (ALK) mutations. The relevance of using C3H mice in this study lies in their utility for modeling human lung cancer and assessing new drug candidates. In addition to genetic predispositions, environmental factors also significantly influence lung cancer development in C3H mice [11].

Consistent with previous research, this study confirmed that I.P administration of benzo(a)pyrene to C3H mice successfully induced lung cancer, characte-rized by tumor formation on the left lung surface and notable pathological changes. Hematological analysis showed a reduction in circulating LYMs, a condition ass-ociated with poor prognosis and immune dysfunction. A previous study by Talebian *et al*. [12] has indicated that patients with advanced NSCLC exhibit high levels of myeloid-derived suppressor cells, which inversely correlate with cluster of differentiation 8+ (CD8+) T-LYM frequencies in peripheral blood. Further analyses through ultrasound imaging and histopathology revealed substantial pathological alterations indicative of lung cancer progression. Ultrasound imaging, a non-invasive diagnostic tool, identified tumors on the left lung surface as deep gaps in the lung parenchyma using the brightness mode (B) acquisition system [12].

Pathological examination of the lung tissues demonstrated dysplastic epithelium and fibroelastosis, critical components of lung cancer pathogenesis, particularly in NSCLC. Dysplastic changes in the bronchial epithelium, considered precursors to malignant transformation, are linked to risk factors such as smoking and environmental exposure. These changes are characterized by abnormal cell morphology and increased proliferation, potentially leading to carcinoma *in situ* and invasive cancer [13, 14]. Histological studies have revealed that dysplastic epithelium often accompanies genetic mutations, including p53 mutations, which are more prevalent in smokers and may serve as early detection biomarkers for lung cancer [13]. In addition, dysplastic epithelium is associated with higher expression of proteins such as mucins and inflammatory cytokines, contributing to lung cancer development [14]. The complex inter-action between dysplastic epithelium and fibroelastosis involves fibroblasts in the tumor microenvironment, influencing epithelial-mesenchymal transition and enhancing cancer cell invasiveness [15]. Transforming growth factor-beta is a crucial mediator in this process, promoting fibroblast activation and dysplastic epithelial changes [16].

The study also identified specific lung cancer protein biomarkers not present in healthy animals, including ADAMTS12, abnormal spindle, adducin 3, aGPCRs, agrin, ACIN1, BRAF, hypoxia-induced gene-1, LRRC2, LRRK2, LRCH2, MSS4A, proto-oncogene tyrosine-protein kinase Src, RAB14, SCCA, TIF1α, and BRCA2. In particular, ADAMTS12 enhances cancer cell migration, contributing to the aggressive nature of lung tumors [17, 18]. Spindle abnormalities linked to tobacco smoke carcinogen exposure can disrupt the mitotic spindle apparatus in bronchial epithelial cells, leading to genomic instability and a higher risk of lung cancer [19].

Furthermore, aGPCRs facilitate tumor cell meta-stasis and migration [20]. The agrin protein, identified in the plasma of lung cancer animal models, has been associated with NSCLC progression through its activation of the phosphatidylinositol 3-kinase/Protein B kinase (PI3K/AKT) signaling pathway. Agrin promotes immune evasion and tumor growth by stimulating regulatory T-cells and increasing interleukin-6 secretion [21]. Hypoxia-induced factor-1 alpha is often overexpressed under hypoxic conditions in lung cancer, promoting angiogenesis and tumor growth by enhancing vascular endothelial growth factor transcription [22, 23]. 4 TIF1α is involved in lung cancer cell proliferation and metastasis [24]. Notably, while BRCA1 and BRCA2 mutations are predominantly associated with breast cancer, this study detected BRCA2 protein in the lung cancer model, suggesting a potential, although lower, role of this protein in lung cancer. Approximately 0.79% of primary lung cancer patients harbor BRCA2 mutations, with this prevalence decreasing to 0.5% in advanced NSCLC cases [25].

Overall, the study demonstrated that benzo(a)pyrene induction in C3H mice effectively produced a lung cancer model, offering valuable insights from hematology, imaging, and pathology to proteomics. These findings could improve the management of animal models at risk and potentially reduce the duration required to establish reliable models. While the induced histopathological changes did not perfectly mimic human lung cancer, they still indicated the transformation of normal cells into malignant forms. As previously discussed, the proteomic screening identified proteins with malignant properties (Table 3). Future research should explore the link between lung parenchymal fibroelastosis and early-stage lung cancer development and assess the potential of nat-ural compounds for chemoprevention and therapy in benzo(a)pyrene-induced animal models.

## CONCLUSION

This study successfully developed a robust lung cancer animal model using I.P. administration of benzo(a)pyrene in inbred C3H mice. The model effe-ctively mirrored critical aspects of human lung cancer, particularly NSCLC, as demonstrated by a comprehensive evaluation of hematological parameters, pathological changes, imaging findings, and proteomic biomarker profiling. The study revealed significant pathological alterations in lung tissues, including dysplastic epithelial changes, fibroelastosis, and LYM infiltration, coupled with a reduction in circulating LYMs. Ultrasound imaging highlighted tumor development on the left lung surface, and proteomic analysis identified specific biomarkers associated with lung cancer, such as ADAMTS12, BRAF, BRCA2, and TIF1α. These findings support the relevance of this model in exploring lung cancer mechanisms and assessing new therapeutic strategies.

The primary strength of this study lies in its multifaceted approach, integrating hematology, adva-nced imaging techniques, histopathological asses-sment, and cutting-edge proteomic analysis. The use of inbred C3H mice adds a unique dimension to lung cancer research, broadening the scope of genetic and environmental factor studies. Furthermore, the identification of specific lung cancer biomarkers enhances the model’s potential as a platform for biomarker discovery and therapeutic target validation.

Despite its strengths, this study has several limitations. First, the histopathological features obser-ved in this model did not completely replicate the histology of human lung cancer, which may limit the direct translational application of findings. In addition, the study focused on a relatively short induction period (7 days), which may not fully capture the progression and metastatic behavior of lung cancer. The sample size was also limited, potentially affecting the generalizability of the results. Finally, while proteomic profiling identified relevant biomarkers, the study did not include functional validation of these proteins in lung cancer progression.

Future research should aim to extend the study duration to assess the model’s ability to simulate advanced and metastatic stages of lung cancer. Expan-ding the sample size and incorporating functional studies of identified biomarkers could provide deeper insights into lung cancer biology. Moreover, evaluating the efficacy of potential chemopreventive agents and therapeutic interventions, including natural com-pounds, could contribute to the development of novel treatments. Exploring the interplay between fibroelastosis and lung cancer development at the early stages of tumorigenesis would also be valuable in understanding the pathophysiological mechanisms involved. This model offers a promising foundation for preclinical testing and the development of personalized therapeutic approaches in lung cancer research.

## AUTHORS’ CONTRIBUTIONS

UK: Investigated, edited, reviewed the manuscript, formal analysis, writing original draft. WTW: Data curation, validated the manuscript. DAAN: Methodology, validated, reviewed, and edited the manuscript. DLK: Conceptualized and supervised the manuscript, writing original draft. DA: Software, project administration. WBTS: Visualized the manuscript, data curation. HW: Conceptualized, supervised, reviewed, and edited the manuscript, funding acquisition, writing original draft. All authors have read and approved the final manuscript.
